# Particle Filter for Randomly Delayed Measurements with Unknown Latency Probability

**DOI:** 10.3390/s20195689

**Published:** 2020-10-06

**Authors:** Ranjeet Kumar Tiwari, Shovan Bhaumik, Paresh Date, Thiagalingam Kirubarajan

**Affiliations:** 1 Department of Electrical Engineering, Indian Institute of Technology Patna, Patna 801106, India; shovan.bhaumik@iitp.ac.in; 2 Department of Mathematics, Brunel University London, Uxbridge UB83PH, UK; paresh.date@brunel.ac.uk; 3 Department of Electrical and Computer Engineering, McMaster University, Hamilton, ON L8S4L8, Canada; kiruba@mcmaster.ca

**Keywords:** nonlinear estimation, particle filte, randomly delayed measurements, latency probability

## Abstract

This paper focuses on developing a particle filter based solution for randomly delayed measurements with an unknown latency probability. A generalized measurement model that includes measurements randomly delayed by an arbitrary but fixed maximum number of time steps along with random packet drops is proposed. Owing to random delays and packet drops in receiving the measurements, the measurement noise sequence becomes correlated. A model for the modified noise is formulated and subsequently its probability density function (pdf) is derived. The recursion equation for the importance weights is developed using pdf of the modified measurement noise in the presence of random delays. Offline and online algorithms for identification of the unknown latency parameter using the maximum likelihood criterion are proposed. Further, this work explores the conditions that ensure the convergence of the proposed particle filter. Finally, three numerical examples, one with a non-stationary growth model and two others with target tracking, are simulated to show the effectiveness and the superiority of the proposed filter over the state-of-the-art.

## 1. Introduction

State estimation for nonlinear discrete-time stochastic systems has received considerable attention from researchers because of its application in various fields of science, including navigation and localization [1,2], surveillance [3], agriculture [4], econometrics [5], and meteorology [6], for example. The Bayesian approach [7] gives a recursive relationship for the computation of the posterior probability density functions (pdf) of the unobserved states. But the computation of the posterior pdf in case of a nonlinear system is often numerically intractable, and hence suboptimal approximations of these pdf are often used. The particle filter (PF) is a powerful sequential Monte Carlo method under the Bayesian framework to solve nonlinear and non-Gaussian estimation problems by approximating the posterior pdf empirically [8]. The particle filter often outperforms other approximate Bayesian filters such as the extended Kalman filter (EKF) and the grid-based filters in solving nonlinear state estimation problems [9]. However, most works on the EKF [10] and as well as on the traditional PF [8,9,11] typically assume that measurements are available at each time step without any delay. In practice, in many aerospace and underwater target tracking [12], control [13] and communication [14] subsystems, random delays in receiving the measurements are inevitable. These delays, usually caused by the limitations of the network channel, need to be accounted for while designing the state estimator.

In the literature, the random delays have been addressed in the context of linear estimators [15,16,17,18,19,20]. A linear networked estimator is proposed in Reference [21] to tackle irregularly-spaced and delayed measurements in a multisensor environment. On the other hand, the research on random delays and packet drops in nonlinear state estimation is limited. In Reference [22] and Reference [23], improved versions of the EKF and the unscented Kalman filter (UKF) are proposed for one-time step and two-time step randomly delayed measurements. In Reference [24], quadrature filters have been modified to solve nonlinear filtering problem with one-step randomly delayed measurements. In Reference [25], the cubature Kalman filter (CKF) [26] is used to tackle one-step randomly delayed measurements for nonlinear systems. In Reference [27], a methodology to solve nonlinear estimation problems with multi-step randomly delayed measurements is proposed. However, all these non-linear filters are restricted to Gaussian approximations. Moreover, they assume that the latency probability of delayed measurements is known. In Reference [28] and Reference [29], a modified PF that deals with one-step randomly delayed measurement with unknown latency probability and a PF for multi-step randomly delayed measurements with a known latency probability are presented, respectively. In References [30,31], the estimation of the unknown latency parameter with one-step randomly delayed measurements is addressed using data log-likelihood function within the Expectation-Maximization (EM) framework. However, none of these works considered the presence of random packet drops. Further, $H_{\infty }$ filtering techniques are used to tackle network-induced delays and packet drops in References [32,33,34].

The specific contributions of this paper to the state-of-the art, specifically over References [28,29,30] are as follows: (i) We propose a new PF with an explicit expression for the importance weight for randomly delayed measurements of any number of time steps with an unknown latency probability and in the presence of packet drops. In Reference [29], the design of a PF with multi-step randomly delayed measurements is addressed. However, the latency probability is assumed to be known and packet drop is not considered while deriving the expression for the importance weight. (ii) The latency parameter for the random delays and packet drops is assumed to be unknown and we present a method to estimate it both in offline and online manners by maximizing the likelihood function. The sequential Monte Carlo (SMC) method is used here to approximate the likelihood function in the presence of randomly delayed measurements of any number of time steps and packet drops. In References [28,30,31] the latency probability for the measurements with random delays of maximum one step is estimated without considering packet drops. Moreover, while Gaussian approximation is used in the E-steps of the EM framework in References [30,31], the SMC approximation is used in Reference [28], but only for measurements with random delays of maximum one time step and without considering any packet drops. (iii) Due to presence of random delays and packet drops, the measurement noise sequence at different time steps becomes correlated. This work first formulates the modified noise model and derives a general pdf for the modified noise. The proposed PF is then developed using the pdf of the modified measurement noise. In References [25,35], randomly delayed measurements with the correlated measurement noise for a nonlinear system is addressed. However, they have considered a maximum delay of one time step and used the Gaussian approximation to develop the filtering algorithm.

Finally, with the help of three numerical examples, the effectiveness and the superiority of the proposed PF are demonstrated in comparison with the state-of-the-art algorithms. Table 1 lists the features of the previous works and of the proposed work.

The rest of this paper is organized as follows. The problem statement is defined in Section 2. In Section 3, the modified PF is proposed and its convergence is discussed. Section 4 deals with the estimation of the unknown latency probability. In Section 5, simulation results are presented to demonstrate the superiority of the proposed PF. Finally, in Section 6, some conclusions are discussed.

## 2. Problem Statement

Consider a nonlinear dynamic system that can be described by the following equations:

(1) $$State\;equation:\;x_{k}=f_{k-1}\left(x_{k-1},k-1\right)+q_{k-1},$$

(2) $$Measurement\;equation:\;z_{k}=h_{k}\left(x_{k},k\right)+v_{k},$$

where $x_{k}\in {ℜ}^{n_{x}}$ denotes the state vector of the system and $z_{k}\in {ℜ}^{n_{z}}$ is the measurement at any discrete time k∈(0,1,⋯), while $q_{k-1}\in {ℜ}^{n_{x}}$ and $v_{k}\in {ℜ}^{n_{z}}$ are mutually independent white noises with arbitrary but known pdf. Here, we consider the case where actual measurement received at a particular time step may be a randomly delayed measurement from a previous time step. This delay (in number of integer time steps) can be between 0 and *N* at the *k*th sampling instant. If any measurement gets delayed by more than *N* steps, that measurement is discarded and no measurement is received at the estimator. Here, *N* is the maximum (in number of integer time steps) delay that is determined as discussed in Section 3.2 and Section 3.3.

To model delayed measurements at the *k*th instant, we choose the independent and identically distributed Bernoulli random numbers $\beta_{k}^{i}$(i=1,2,⋯,N+1) that take values either 0 or 1 with an unknown probability $P\left(\beta_{k}^{i}=1\right)=p=E\left[\beta_{k}^{i}\right]$ and $P(\beta_{k}^{i}=0)=1-p$, where *p* is the unknown latency parameter. If $y_{k}$ is the measurement received at the *k*th instant [27], then

(3) $$\begin{array}{cc} y_{k} & =\left(1-\beta_{k}^{1}\right)z_{k}+\beta_{k}^{1}\left(1-\beta_{k}^{2}\right)z_{k-1}+\beta_{k}^{1}\beta_{k}^{2}\left(1-\beta_{k}^{3}\right)z_{k-2}+\cdots +\prod_{i=1}^{N}\beta_{k}^{i}\left(1-\beta_{k}^{N+1}\right)z_{k-N}+(1-\left(1-\beta_{k}^{1}\right)\\ & -\beta_{k}^{1}\left(1-\beta_{k}^{2}\right)-\cdots -\prod_{i=1}^{N}\beta_{k}^{i}\left(1-\beta_{k}^{N+1}\right))y_{k-1},\\ \; & =\sum_{j=0}^{N}\alpha_{k}^{j}z_{k-j}+\left(1-\sum_{j=0}^{N}\alpha_{k}^{j}\right)y_{k-1};\;k\geq 2, \end{array}$$

where

(4) $$\alpha_{k}^{j}=\prod_{i=0}^{j}\beta_{k}^{i}\left(1-\beta_{k}^{j+1}\right).$$

Here, $\beta_{k}^{0}$ is considered to be 1. A measurement received at the *k*th time instant is *j* step delayed if $\alpha_{k}^{j}=1$. Additionally, at time instant *k*, at most one of $\alpha_{k}^{j}\left(0\leq j\leq N\right)$ can be 1. If all $\alpha_{k}^{j}$ are zeros, the estimator buffer keeps the measurement $y_{k-1}$ received at the previous step. This results in the loss of a measurement (packet drop) when that measurement is delayed by more than *N* steps due to buffer-size limitation.

**Remark** **1.** *Bernoulli random variable $\beta_{k}^{i}$ and its function $\alpha_{k}^{j}$ are used to represent the real-time randomness of delays in measurements in practical systems [15]. Inclusion of the possibility that at a time step k no measurement may be received (when the delay is longer than N steps) corresponds to the practical limit on the buffer size in real systems. In our algorithm, if a received measurement matches with any of the measurements in the buffer, it is discarded.*

**Remark** **2.** *The latency parameter of received measurements, p, that is, the mean of random variable $\beta_{k}^{i}$ is unknown in real systems. Contrary to the assumption of single-step delay in Reference [28], the unknown latency probability in this paper is for arbitrary step delays along with the packet drops due to buffer limitation.*

Further, since both the ideal measurement and the measurement noise are modified in (3), $y_{k}$ needs to be rewritten for its subsequent use in characterizing the densities. Hereafter in this article, $h_{k}\left(x_{k},k\right)$ and $f_{k}\left(x_{k},k\right)$ will be written as $h_{k}\left(x_{k}\right)$ and $f_{k}\left(x_{k}\right)$ respectively, for brevity. Then, (3) can be restructured as

(5) $$y_{k}=G1_{k}\left(h_{k-j}\left(x_{k-j}\right),\alpha_{k}^{j}\right)+G2_{k}\left(G1_{k-1}\left(\cdot \right),\alpha_{k}^{j},G2_{k-1}\left(\cdot \right)\right)+v_{k}^{\prime };\;k\geq 2\;\mathrm{and}\;0\leq j\leq N.$$

Here, $G1_{k}\left(h_{k-j}\left(x_{k-j}\right),\alpha_{k}^{j}\right)$ and $G2_{k}\left(G1_{k-1}\left(\cdot \right),\alpha_{k}^{j},G2_{k-1}\left(\cdot \right)\right)$ denote the ideal measurement parts (without any noise) of $y_{k}$ from the non-delayed measurements $z_{k-N:k}$ and the previous step measurement $y_{k-1}$, respectively, and are defined as

(6) $$G1_{k}\left(h_{k-j}\left(x_{k-j}\right),\alpha_{k}^{j}\right)=\sum_{j=0}^{N}\alpha_{k}^{j}h_{k-j}\left(x_{k-j}\right);\;k\geq 2$$

(7) $$G2_{k}\left(G1_{k-1}\left(\cdot \right),\alpha_{k}^{j},G2_{k-1}\left(\cdot \right)\right)=\left(1-\sum_{j=0}^{N}\alpha_{k}^{j}\right)\left(G1_{k-1}\left(\cdot \right)+G2_{k-1}\left(\cdot \right)\right);\;k\geq 2,$$

where $G1_{1}\left(\cdot \right)=h_{1}\left(x_{1}\right)$ and $G2_{1}\left(\cdot \right)=0$. Again, $v_{k}^{\prime }$ is the additive measurement noise in (3) defined as

(8) $$v_{k}^{\prime }=\sum_{j=0}^{N}\alpha_{k}^{j}v_{k-j}+\left(1-\sum_{j=0}^{N}\alpha_{k}^{j}\right)v_{k-1}^{\prime };\;k\geq 2,$$

where $v_{k-1}^{\prime }$ is the noise received along with observation $y_{k-1}$ and $v_{1}^{\prime }=v_{1}$.

Now, the objective is to develop a PF algorithm for the system in (1) with measurement model (3) that assumes the knowledge of latency probability *p*. Additionally, we propose offline as well as online algorithms to estimate *p*.

## 3. Modified Particle Filter for Randomly Delayed Measurements

### 3.1. Particle Filter

In a sequential importance sampling filter, the posterior probability density function $P(x_{0:k}|z_{1:k})$ is replaced by its equivalent series of weighed particles, which can be represented as [9]

(9) $$\hat{P}\left(x_{0:k}|z_{1:k}\right)=\sum_{i=1}^{N_{s}}w_{k}^{i}\delta \left[x_{0:k}-x_{0:k}^{i}\right],$$

where particles ${\left\{x_{0:k}^{i}\right\}}_{i=1}^{N_{s}}$ are drawn from a proposal density $q(x_{0:k}|z_{1:k})$ and then the weights of the particles are chosen using the importance principle. The unnormalized importance weight of the *i*th particle is given by

(10) $$w_{k}^{i}=\frac{P(x_{0:k}^{i}|z_{1:k})}{q(x_{0:k}^{i}|z_{1:k})}.$$

The recursive weight update at each time step is given by

(11) $$\begin{array}{c} P\left(x_{0:k}|z_{1:k}\right)=\frac{P\left(z_{k}|x_{0:k}\right)P\left(x_{0:k}|z_{1:k-1}\right)}{P(z_{k}|z_{1:k-1})}\\ \propto P\left(z_{k}|x_{k}\right)P\left(x_{k}|z_{1:k-1}\right), \end{array}$$

where $P(z_{k}|z_{1:k-1})$ is a normalizing constant. Similarly, the proposal density is assumed to be decomposed as

(12) $$q\left(x_{0:k}|z_{1:k}\right)=q\left(x_{k}|x_{0:k-1},z_{1:k}\right)q\left(x_{0:k-1}|z_{1:k-1}\right).$$

Assuming that the state vector $x_{k}$ follows the Markov process, the importance weight of (10), with the help of (11) and (12), can be written as

$$\begin{array}{c} \begin{array}{cc} w_{k}^{i} & \propto \frac{P\left(z_{k}|x_{k}^{i}\right)P\left(x_{k}^{i}|x_{k-1}^{i}\right)P\left(x_{k-1}^{i}|z_{1:k-1}\right)}{q\left(x_{k}^{i}|x_{0:k-1}^{i},z_{1:k}\right)q\left(x_{0:k-1}^{i}|z_{1:k-1}\right)}\\ \; & =w_{k-1}^{i}\frac{P\left(z_{k}|x_{k}^{i}\right)P\left(x_{k}^{i}|x_{k-1}^{i}\right)}{q(x_{k}^{i}|x_{0:k-1}^{i},z_{1:k})}, \end{array} \end{array}$$

where the predicted density $P(x_{k}^{i}|x_{k-1}^{i})$ and the likelihood density $P(z_{k}|x_{k}^{i})$ can be evaluated using the system model, previously estimated posterior $P(x_{k-1}^{i}|z_{1:k-1})$ and the joint noise density $P(q_{k-1},v_{k}|x_{k-1}^{i})$ [7].

### 3.2. Modified PF for Randomly Delayed Measurements

A recursive computation of the importance weights can be obtained for a nonlinear system with measurement model of (3). From (3), it can be seen that $y_{k}$ is stochastically dependent on the non-delayed measurements $z_{k-N:k}$ and the previous step measurement $y_{k-1}$ and, therefore, we need to relax the standard assumption of $P\left(z_{k}|x_{1:k},z_{1:k-1}\right)=P\left(z_{k}|x_{k}\right)$ as discussed below.

**Assumption** **1.** *The received measurement $y_{k}$, conditionally on $x_{k-N:k}\;\mathrm{and}\;y_{k-1}$, is independent of the state vectors $x_{1:k-N-1}$ and the measurements $y_{1:k-2}$, that is, $P\left(y_{k}|x_{1:k},y_{1:k-1}\right)=P\left(y_{k}|x_{k-N:k},y_{k-1}\right)$.*

**Lemma** **1.** *Recursion equation of the importance weight $w_{k}^{i}$ for model (1) and (3), can be obtained as*

(13) $$w_{k}^{i}=w_{k-1}^{i}\frac{P\left(y_{k}|x_{k-N:k}^{i},y_{k-1}\right)P\left(x_{k}^{i}|x_{k-1}^{i}\right)}{q(x_{k}^{i}|x_{0:k-1}^{i},y_{1:k})},$$

*where $x_{k}^{i}$ is drawn from the proposal density $q(x_{k}|x_{0:k-1}^{i},y_{1:k})$.*

**Proof.**  Using the Bayes’ theorem and assuming that the states do not depend on the future measurements, the proposal density can be decomposed as

(14) $$\begin{array}{c} \begin{array}{c} q\left(x_{0:k}|y_{1:k}\right)=q\left(x_{k}|x_{1:k-1},y_{1:k}\right)q\left(x_{1:k-1}|y_{1:k-1}\right). \end{array} \end{array}$$

Particles $x_{k}^{i}$ and $x_{1:k-1}^{i}$ can be sampled from $q(x_{k}|x_{1:k-1},y_{1:k})$ and $q(x_{1:k-1}|y_{1:k-1})$, respectively. Again, using the Bayes’ rule, the joint pdf, $P(x_{1:k},y_{1:k})$, can be decomposed as follows:

(15) $$\begin{array}{c} \begin{array}{cc} P(x_{1:k},y_{1:k})\; & =P\left(y_{k}|x_{k},x_{1:k-1},y_{1:k-1}\right)P\left(x_{k},x_{1:k-1},y_{1:k-1}\right)\\ \; & =P\left(y_{k}|x_{k},x_{1:k-1},y_{1:k-1}\right)P\left(x_{k}|x_{1:k-1},y_{1:k-1}\right)P\left(x_{1:k-1},y_{1:k-1}\right). \end{array} \end{array}$$

By Assumption 1 and the first-order Markov property of the state vectors, (15) can be rewritten as

(16) $$P\left(x_{1:k},y_{1:k}\right)=P\left(y_{k}|x_{k-N:k},y_{k-1}\right)P\left(x_{k}|x_{k-1}\right)P\left(x_{1:k-1},y_{1:k-1}\right).$$

Using (14) and (16), the importance weight can be written as

(17) $$\begin{array}{c} \begin{array}{cc} w_{k}\; & =\frac{P\left(y_{k}|x_{k-N:k},y_{k-1}\right)P\left(x_{k}|x_{1:k-1}\right)}{q(x_{k}|x_{0:k-1},y_{1:k})}\frac{P(x_{1:k-1},y_{1:k-1})}{q(x_{0:k-1}|y_{1:k-1})}\\ \; & =w_{k-1}\frac{P\left(y_{k}|x_{k-N:k},y_{k-1}\right)P\left(x_{k}|x_{1:k-1}\right)}{q(x_{k}|x_{0:k-1},y_{1:k})}. \end{array} \end{array}$$

Now, with the help of (17), $w_{k}^{i}$ can be finally written as (13). □

Now, the predicted density $P(x_{k}^{i}|x_{k-1}^{i})$ and the likelihood density $P(y_{k}|x_{k-N:k}^{i},y_{k-1})$ can be characterized using the joint noise density $P(q_{k-1},v_{k}^{\prime }|x_{k-1})$ [7,36]. As given in Section 2, $q_{k-1}$ and $v_{k}$ are independent noise processes with $E\left[q_{k-1-i}{\left(v_{k-j}\right)}^{⊤}\right]=E\left[q_{k-1-i}\right]E\left[v_{k-j}\right]=0$, for all integer values of *i* and *j*. Hence, using the independence property and (8), it can be shown that $q_{k-1}$ and the modified measurement noise $v_{k}^{\prime }$ are also independent. Therefore, assuming $q_{k-1}$ and $v_{k}^{\prime }$ are independent of the previous state $x_{k-1}$, we can decompose the joint noise density as $P\left(q_{k-1},v_{k}^{\prime }|x_{k-1}\right)=P\left(q_{k-1}\right)P\left(v_{k}^{\prime }\right)$. Moreover, given that the pdf of $q_{k-1}$ is known, $P(x_{k}^{i}|x_{k-1}^{i})$ can be evaluated, whereas the pdf of $v_{k}^{\prime }$ is unknown and needs to be calculated for the evaluation of the likelihood.

Further, for the computation of the likelihood density $P(y_{k}|x_{k-N:k},y_{k-1})$, the probability related to the number of random delays in the received measurement needs to be evaluated. Note that the probability of a received measurement being delayed by *j* time steps, at any instant *k*, is [27] $P\left(\alpha_{k}^{j}=1\right)=p^{j}\left(1-p\right)$, 0≤j≤N. Note that as the number of delay steps (*j*) increases, the associated probability ($P(\alpha_{k}^{j}=1)$) decreases. The probability that the estimator receives $y_{k-1}$ at the *k*th instant of time is [27] $P\left(\sum_{j=0}^{N}\alpha_{k}^{j}=0\right)=p^{N+1}$. It can be observed that for a high value of *p*, *N* should be kept sufficiently large to reduce the probability of a packet being lost.

**Lemma** **2.** *The likelihood density $P(y_{k}|x_{k-N:k}^{i},y_{k-1})$ can be computed recursively as*

(18) $$\begin{array}{c} \begin{array}{c} P\left(y_{k}|x_{k-N:k}^{i},y_{k-1}\right)=\sum_{j=0}^{N}p^{j}\left(1-p\right)P_{v_{k-j}}\left(y_{k}-h_{k-j}\left(x_{k-j}^{i}\right)\right)+p^{N+1}P\left(y_{k-1}|x_{k-1-N:k-1}^{i},y_{k-2}\right), \end{array} \end{array}$$

*where $P_{v_{k-j}}\left(\cdot \right)$ represents the pdf of the measurement noise $v_{k-j}$.*

**Proof.**  Let $\gamma_{k}$ be a Bernoulli random variable that denotes the event that a measurement is received (with a step delay between 0 and *N*). The probability that a measurement is received with a delay less than or equal to *N* steps, is

(19) $$P\left(\gamma_{k}=1\right)=P\left(\sum_{j=0}^{N}\alpha_{k}^{j}=1\right)=\sum_{j=0}^{N}p^{j}\left(1-p\right).$$

The probability that no measurement is received and the estimator keeps measurement $y_{k-1}$, is

(20) $$P\left(\gamma_{k}=0\right)=P\left(\sum_{j=0}^{N}\alpha_{k}^{j}=0\right)=p^{N+1}.$$

Now, as the modified likelihood density should be characterized by the pdf of the modified measurement noise $v_{k}^{\prime }$, using (5), we have

(21) $$P\left(y_{k}|x_{k-N:k},y_{k-1}\right)=P_{v_{k}^{\prime }}\left(y_{k}-G1_{k}\left(h_{k-j}\left(x_{k-j}\right),\alpha_{k}^{j}\right)-G2_{k}\left(G1_{k-1}\left(\cdot \right),\alpha_{k}^{j},G2_{k-1}\left(\cdot \right)\right)\right),$$

where $P_{v_{k}^{\prime }}\left(\cdot \right)$ denotes the pdf of $v_{k}^{\prime }$. Further, assuming that $v_{k}^{\prime }$, conditionally on $v_{k-N:k}$ and $v_{k-1}^{\prime }$, is an independent noise sequence over time, it can be calculated using the known pdf of $v_{k-N:k}$ and the intermediate Bernoulli random variable $\gamma_{k}$ as follows:

(22) $$\begin{array}{c} \begin{array}{cc} P(v_{k}^{\prime }) & =\int P\left(v_{k}^{\prime },\gamma_{k}\right)d\gamma_{k}\\ \; & =\sum_{\gamma_{k}=0}^{1}P\left(v_{k}^{\prime }|\gamma_{k}\right)P\left(\gamma_{k}\right)\\ \; & =P\left(v_{k}^{\prime }|\gamma_{k}=1\right)P\left(\gamma_{k}=1\right)+P\left(v_{k}^{\prime }|\gamma_{k}=0\right)P\left(\gamma_{k}=0\right)\\ & \left(\mathrm{using}\;(19)\;\mathrm{and}\;(20)\right)\\ \; & =\sum_{j=0}^{N}p^{j}\left(1-p\right)\times P\left(v_{k}^{\prime }|\gamma_{k}=1\right)+p^{N+1}\times P\left(v_{k}^{\prime }|\gamma_{k}=0\right). \end{array} \end{array}$$

Using the expression for $v_{k}^{\prime }$ in (8), if $\gamma_{k}=1$ (i.e., $\alpha_{k}^{j}=1$ for any *j*), we can write $v_{k}^{\prime }=v_{k-j}$ and

(23) $$P\left(v_{k}^{\prime }|\gamma_{k}=1\right)=P\left(v_{k-j}\right).$$

Similarly, when $\gamma_{k}=0$ (i.e., $\sum_{j=0}^{N}\alpha_{k}^{j}=0$), the measurement noise $v_{k}^{\prime }=v_{k-1}^{\prime }$ and we have

(24) $$P\left(v_{k}^{\prime }|\gamma_{k}=0\right)=P\left(v_{k-1}^{\prime }\right).$$

Substituting (23) and (24) into (22) and then, (22) and (21) results in

(25) $$\begin{array}{ll} P(y_{k}|x_{k-N:k},y_{k-1}) & =\sum_{j=0}^{N}p^{j}\left(1-p\right)P_{v_{k-j}}\left(\left(y_{k}-G1_{k}\left(h_{k-j}\left(x_{k-j}\right),\alpha_{k}^{j}\right)-G2_{k}\left(G1_{k-1}\left(\cdot \right),\alpha_{k}^{j},G2_{k-1}\left(\cdot \right)\right)\right){|}_{\gamma_{k}=1}\right)\\ & +p^{N+1}P_{v_{k-1}^{\prime }}\left(\left(y_{k}-G1_{k}\left(h_{k-j}\left(x_{k-j}\right),\alpha_{k}^{j}\right)-G2_{k}\left(G1_{k-1}\left(\cdot \right),\alpha_{k}^{j},G2_{k-1}\left(\cdot \right)\right)\right){|}_{\gamma_{k}=0}\right). \end{array}$$

By definition, $\gamma_{k}=\Sigma_{j=0}^{N}\alpha_{k}^{j}$. Again, using (6), we can write $\left(y_{k}-G1_{k}\left(\cdot \right)-G2_{k}\left(\cdot \right)\right){|}_{\gamma_{k}=1}$$=y_{k}-h_{k-j}\left(x_{k-j}\right),\;$0≤j≤N. Similarly, using (3) and (7), we have $\left(y_{k}-G1_{k}\left(\cdot \right)-G2_{k}\left(\cdot \right)\right){|}_{\gamma_{k}=0}=y_{k-1}-G1_{k-1}\left(\cdot \right)-G2_{k-1}\left(\cdot \right)$. Now, rewriting (25) for particle *i*, we have

$$\begin{array}{l} \begin{array}{ll} P(y_{k}|x_{k-N:k}^{i},y_{k-1}) & =\sum_{j=0}^{N}p^{j}\left(1-p\right)P_{v_{k-j}}\left(y_{k}-h_{k-j}\left(x_{k-j}^{i}\right)\right)+p^{N+1}P_{v_{k-1}^{\prime }}\left(y_{k-1}-G1_{k-1}\left(\cdot \right)-G2_{k-1}\left(\cdot \right)\right),\\ & \left(\mathrm{applying}\;(21)\;\mathrm{for}\;k:=k-1\;\mathrm{to}\;\mathrm{the}\;\sec\mathrm{ond}\;\mathrm{term}\;\mathrm{on}\;\mathrm{the}\;\mathrm{right}\;\mathrm{hand}\;\mathrm{side}\right)\\ \; & =\sum_{j=0}^{N}p^{j}\left(1-p\right)P_{v_{k-j}}\left(y_{k}-h_{k-j}\left(x_{k-j}^{i}\right)\right)+p^{N+1}P\left(y_{k-1}|x_{k-1-N:k-1}^{i},y_{k-2}\right). \end{array} \end{array}$$

□

**Remark** **3.** *Note that (18) is similar to the sum of the product densities used in the probabilistic data association (PDA) algorithm [37,38]. Further, it can be observed that if there are no random delays and packet drops in the received measurements (i.e., N=0 and p=0), (18) reduces to $P_{v_{k}}\left(z_{k}-h_{k}\left(x_{k}^{i}\right)\right)$, the expression for the likelihood density of the standard PF.*

**Figure 1:**
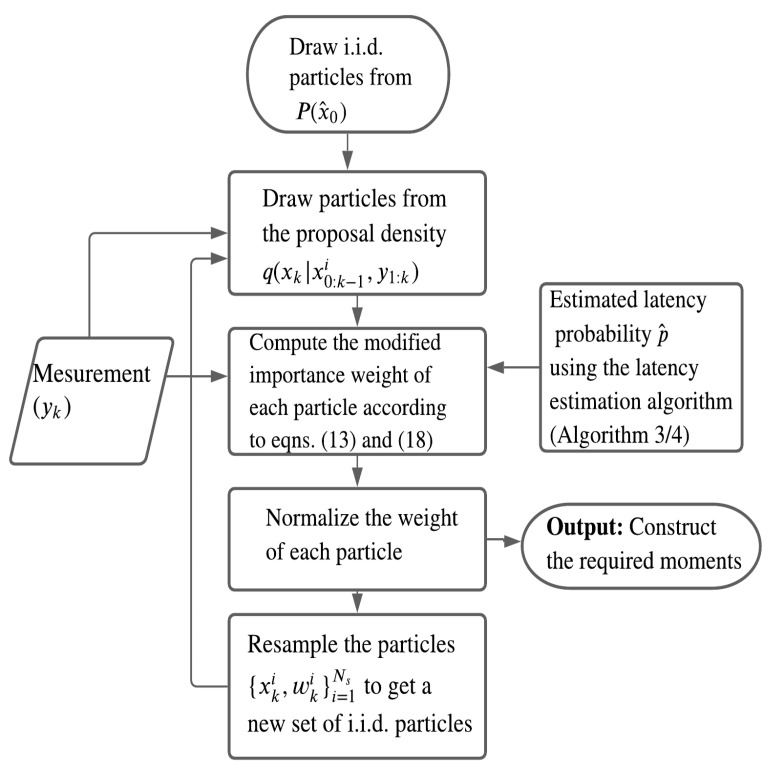
Flow diagram for PF with the modified importance weight.

In this work, to mitigate the effect of degeneracy on the importance weights of particles, the resampling is carried out at each step. A general proposal density $q(x_{k}|x_{0:k-1},y_{1:k})$ obtained from a nonlinear filter such as the EKF or the UKF [39], can be used to draw samples. However, the predicted density $P(x_{k}|x_{k-1})$ is a common choice to implement the sequential importance resampling (SIR) PF [9] despite the fact that the current measurement is not used to locate new samples. Note that the current measurement in this work is randomly delayed and is unaccounted for in the proposal density, which may not necessarily push the sampled particles towards higher likelihood regions. On the other hand, $P(x_{k}|x_{k-1})$ includes the measurements up to the last time-step that have been corrected with the modified importance weight. The steps to implement a standard SIR filter [9] with the modified importance weight for this work are outlined in Algorithm 1. It uses a standard resampling technique, for example, multinomial [40] or, systematic [9] to suppress the particles with negligible weight. A flow diagram to obtain the modified PF estimate is shown in Figure 1.   
**Algorithm 1.** Modified particle filter. $\left[{\left\{x_{k}^{i},w_{k}^{i}\right\}}_{i=1}^{N_{s}}\right]:=\mathrm{SIR}\left[{\left\{x_{k-N:k}^{i},w_{k-1}^{i}\right\}}_{i=1}^{N_{s}},\hat{p},y_{k}\right]$*for*$i=1:N_{s}$–Draw$x_{k}^{i}∼q\left(x_{k}|x_{0:k-1}^{i},y_{1:k}\right)$–Compute the importance weight $w_{k}^{i}$ according to (13) and (18)–Normalize the importance weight:$\;w_{k}^{i}:=w_{k}^{i}/\mathrm{SUM}\left[{\left\{w_{k}^{i}\right\}}_{i=1}^{N_{s}}\right]$end forResample the particles at each step–$\left[{\left\{x_{k}^{i},w_{k}^{i}\right\}}_{i=1}^{N_{s}}\right]:=\mathrm{RESAMPLE}\left[{\left\{x_{k}^{i},w_{k}^{i}\right\}}_{i=1}^{N_{s}}\right]$

### 3.3. Convergence of the PF for Randomly Delayed Measurements

In this subsection, we explore the conditions for the convergence of the modified PF derived for randomly delayed measurements. A PF is said to be converging if its empirical approximation follows a mean square error of order $1/N_{s}$ at each step of filtering [8]. The prime requisite for simple convergence is that likelihood function $P(z_{k}|\cdot )$ should be upper bounded, that is, $∥P(z_{k}|\cdot )∥<\infty$, for all $x_{k}\in {ℜ}^{n_{x}}$ [8]. The following lemma is an extension of the results in Reference [8] for our case.

**Lemma** **3.** *If the likelihood function*

(26) $$∥P\left(y_{k}|x_{k-N:k},y_{k-1}\right)∥<\infty ,\;\forall x_{k-N:k}\in {ℜ}^{n_{x}}\;and\;y_{k}\in {ℜ}^{n_{z}},$$

*then, ∀k≥N, there exists $c_{k/k}$ independent of $N_{s}$, such that for any $\Phi \in B({ℜ}^{n_{x}\times \left(k+1\right)})$*

(27) $$E\left[{\left(\frac{1}{N_{s}}\sum_{i=1}^{N_{s}}\Phi \left(x_{0:k}^{i}\right)-\int \Phi \left(x_{0:k}\right)P\left(dx_{0:k}|y_{1:k}\right)\right)}^{2}\right]\leq c_{k/k}\frac{{∥\Phi ∥}^{2}}{N_{s}},$$

*where $x_{0:k}^{i}$ are the unweighted particles obtained using the modified PF algorithm.*

**Proof.**  Attributing to the random delays and packet drops in measurements, we investigate the impact of the modified likelihood density on the simple convergence of the particle filter that is otherwise converging with non-delayed measurements. That is, $∥P\left(z_{k}|x_{k}\right)∥<\infty ,\;\forall x_{k}\in {ℜ}^{n_{x}}\;\mathrm{and}\;z_{k}\in {ℜ}^{n_{z}}$, is given. Now, using (2), we can write

$$P\left(z_{k}|x_{k}\right)=P_{v_{k}}\left(z_{k}-h_{k}\left(x_{k}\right)\right).$$

Thus, the pdf of noise $v_{k}$, that is, $P_{v_{k}}\left(z_{k}-h_{k}\left(x_{k}\right)\right)$, is bounded for all its real-valued inputs. Rearranging the terms of (18) on the both sides, we have

(28) $$\begin{array}{c} P\left(y_{k}|x_{k-N:k},y_{k-1}\right)-p^{N+1}P\left(y_{k-1}|x_{k-1-N:k-1}\right)=\sum_{j=0}^{N}p^{j}\left(1-p\right)P_{v_{k-j}}\left(y_{k}-h_{k-j}\left(x_{k-j}\right)\right). \end{array}$$

As $v_{k}$ is a stationary process, its pdf is not affected by time shift, that is, if $P_{v_{k}}\left(\cdot \right)$ is bounded, $P_{v_{k-j}}\left(\cdot \right)$ must also be bounded. Again, since $p^{j}\left(1-p\right)<1$ for all values of *j*, we can write

(29) $$\begin{array}{c} \sum_{j=0}^{N}p^{j}\left(1-p\right)P_{v_{k-j}}\left(y_{k}-h_{k-j}\left(x_{k-j}\right)\right)\leq \left(N+1\right)P_{v_{k-j}}\left(y_{k}-h_{k-j}\left(x_{k-j}\right)\right). \end{array}$$

Given that *N* is a finite number, (28) and (29) can be used to establish (26). Now, results of Reference [8] can be used to verify (27). □

Theorem 3 of Reference [8] suggests that $c_{k|k}$ is independent of the number of particles $N_{s}$, but represents the dependency of mixed dynamics of the system on the initial conditions or past values. That is, if the optimal filter associated with the system dynamics has a long memory, $c_{k|k}$ will continue to increase the mean square error with each step. In our case, the filter does have a memory due to arbitrary delays in measurements. Therefore, to avoid the large mean square error of convergence, the value of *N* should be kept small. On the other hand, to counter the increase in the value of $c_{k|k}$, number of particles $N_{s}$ needs to be large.

## 4. Identification of Latency Probability

In practice, when a set of randomly delayed measurements is given, we may not know channel properties and its random parameters. Hence, the latency probability that is required to design the filter may be unknown. Here, we use the ML criterion to identify the unknown latency probability for received measurements. This method involves the maximization of the joint density $P_{p}\left(y_{1:m}\right)$ of the received measurements, which is a function of latency parameter *p* represented by [28]

(30) $$\hat{p}=\arg\;\max_{p\in [0,1]}P_{p}\left(y_{1},\cdots ,y_{m}\right),$$

where *m* is the number of measurements used for the identification of parameter *p* and $\hat{p}$ is the estimated value of *p*. Now, we assume that the first received measurement $y_{1}$ is independent of parameter *p* and is equal to $z_{1}$. Again, using the Bayes’ theorem the above joint pdf can be reformulated as

(31) $$P_{p}\left(y_{1},\cdots ,y_{m}\right)=P\left(y_{1}\right)\prod_{k=2}^{m}P_{p}\left(y_{k}|y_{1:k-1}\right).$$

For computational simplicity the above maximization of likelihood is expressed in terms of the log-likelihood (LL). The LL of (31) can be formulated as

(32) $$\begin{array}{c} \begin{array}{cc} L_{p}\left(y_{1:m}\right) & =\log P_{p}\left(y_{1:m}\right)\\ \; & =\log P\left(y_{1}\right)+\sum_{k=2}^{m}\log P_{p}\left(y_{k}|y_{1:k-1}\right), \end{array} \end{array}$$

where $L_{p}\left(y_{1:m}\right)$ is the LL function of the received measurements. Now, to solve the maximization problem of (31), first the computation of likelihood $P_{p}\left(y_{k}|y_{1:k-1}\right)$ and then the maximization of $L_{p}\left(y_{1:m}\right)$ need to be carried out.

### 4.1. Computation of Likelihood Density

State likelihood can be used to compute $P_{p}\left(y_{k}|y_{1:k-1}\right)$ with the help of the SMC approximation [41]. We can express the likelihood density $P_{p}\left(y_{k}|y_{1:k-1}\right)$ as the marginal density of a joint pdf that includes delayed measurement and previous states that are correlated using the Bayes’ theorem and Assumption 1 as follows:

(33) $$\begin{array}{c} \begin{array}{cc} P_{p}\left(y_{k}|y_{1:k-1}\right)\; & =\int ...\int P_{p}\left(y_{k},x_{k-N:k}|y_{1:k-1}\right)dx_{k}\cdots dx_{k-N}\\ \; & =\int ...\int P_{p}\left(y_{k}|x_{k-N:k},y_{1:k-1}\right)P_{p}\left(x_{k-N:k}|y_{1:k-1}\right)dx_{k}\cdots dx_{k-N}\\ \; & =\int ...\int P_{p}\left(y_{k}|x_{k-N:k},y_{k-1}\right)P_{p}\left(x_{k-N:k}|y_{1:k-1}\right)dx_{k}\cdots dx_{k-N}. \end{array} \end{array}$$

Using Bayes’ rule, the joint state prior $P_{p}\left(x_{k-N:k}|y_{1:k-1}\right)$ can be decomposed as

$$\begin{array}{c} \begin{array}{c} P_{p}\left(x_{k-N:k}|y_{1:k-1}\right)=P_{p}\left(x_{k}|x_{k-N:k-1},y_{1:k-1}\right)P_{p}\left(x_{k-N:k-1}|y_{1:k-1}\right) \end{array} \end{array}$$

Since the state vectors follow the first-order Markov property, using the chain rule and (9), we can write the joint pdf as

(34) $$\begin{array}{c} \begin{array}{cc} P_{p}\left(x_{k-N:k}|y_{1:k-1}\right) & =P\left(x_{k}|x_{k-1}\right)P_{p}\left(x_{k-1}|x_{k-2},y_{k-1}\right)\cdots P_{p}\left(x_{k-N}|x_{k-N-1},y_{k-N}\right),\\ \; & \approx \frac{1}{N_{s}}\sum_{i=1}^{N_{s}}\delta \left[x_{k}-x_{k}^{i}\right]\delta \left[x_{k-1}-x_{k-1}^{i}\right]\cdots \delta \left[x_{k-N}-x_{k-N}^{i}\right], \end{array} \end{array}$$

where $x_{k-N}^{i},x_{k-N+1}^{i},\cdots ,$ and $x_{k}^{i}$ are the unweighted particles and drawn from $P_{p}\left(x_{k-N}|x_{k-N-1},y_{k-N}\right),P_{p}\left(x_{k-N+1}|x_{k-N},y_{k-N+1}\right),\cdots ,$ and $P(x_{k}|x_{k-1})$, respectively. The accuracy of the SMC approximation in this case depends on the choice of the proposal density, the number of particles and the value of *N*. This would be equivalent to that of the modified SIR as discussed in Section 3.3. Now, substituting (34) into (33), $P_{p}\left(y_{k}|y_{1:k-1}\right)$ can be computed as

(35) $${\hat{P}}_{p}\left(y_{k}|y_{1:k-1}\right)=\frac{1}{N_{s}}\sum_{i=1}^{N_{s}}P_{p}\left(y_{k}|x_{k}^{i},\cdots ,x_{k-N}^{i},y_{k-1}\right),$$

where $P_{p}\left(y_{k}|x_{k-N:k}^{i},y_{k-1}\right)$ is given in (18). Algorithm 2 illustrates the steps to compute the LL function.
**Algorithm 2.** Computation of log-likelihood function.[$L_{p},{\left\{x_{k}^{i},w_{p,k}^{i}\right\}}_{i=1}^{N_{s}}]:=\mathrm{LL}[L_{p},{\left\{x_{k-N:k-1}^{i},w_{p,k-1}^{i}\right\}}_{i=1}^{N_{s}},y_{k}$]*for* ($i=1:N_{s}$)
–Draw $x_{k}^{i}∼q\left(x_{k}|x_{0:k-1}^{i},y_{1:k}\right)$–Assign particle a weight:
$w_{p,k}^{i}=w_{p,k-1}^{i}\frac{P_{p}\left(y_{k}|x_{k-N:k}^{i},y_{k-1}\right)P\left(x_{k}^{i}|x_{k-1}^{i}\right)}{q(x_{k}^{i}|x_{0:k-1}^{i},y_{1:k})}$end forCompute the LL function:
$$L_{p}:=L_{p}+\log\left(\frac{1}{N_{s}}\sum_{i=1}^{N_{s}}P_{p}\left(y_{k}|x_{k-N:k}^{i},y_{k-1}\right)\right)$$*for* ($i=1:N_{s}$)
–Normalize the importance weight: $w_{p,k}^{i}:=w_{p,k}^{i}/\mathrm{SUM}\left[{\left\{w_{p,k}^{i}\right\}}_{i=1}^{N_{s}}\right]$end forResample the drawn particles at each step:
 $\left[{\left\{x_{k}^{j},w_{p,k}^{j}\right\}}_{j=1}^{N_{s}}\right]:=\mathrm{RESAMPLE}\left[{\left\{x_{k}^{i},w_{p,k}^{i}\right\}}_{i=1}^{N_{s}}\right]$

### 4.2. Maximization of Log-Likelihood Function

Substituting (35) into (32), we can rewrite the LL function in (32) as

(36) $${\hat{L}}_{p}\left(y_{1:m}\right)=\sum_{k=2}^{m}\log\left(\frac{1}{N_{s}}\sum_{i=1}^{N_{s}}P_{p}\left(y_{k}|x_{k-N:k}^{i},y_{k-1}\right)\right),$$

where $y_{1}$ is independent of parameter *p* and can be neglected in the maximization of the likelihood density. Equation (36) can be maximized numerically over p∈[0,1].

There are two options for the numerical search of the latency parameter: offline and online identification. In the offline method, we can use more measurements for higher parameter estimation accuracy. A higher value of *m* and smaller steps (sl) for *p* can yield a more accurate estimate of the latency probability, at the expense of higher computational burden. The offline algorithm can only be started after the first *m* measurements and it can be repeated with further measurements to improve the estimate. Algorithm 3 outlines the steps for offline identification.

In case of online identification, the latency probability is estimated at each time step and the estimated value of the parameter is used in the proposed PF algorithm. The running average of the estimated values at each step can be evaluated to improve the accuracy of the identified parameter. Algorithm 4 outlines the online method.
**Algorithm 3.** Offline identification of latency probability.[$L_{max},\hat{p}]:=\mathrm{OFFLINE}[{\left\{x_{k-N:k}^{i},w_{k-1}^{i}\right\}}_{i=1}^{N_{s}},y_{1:m}$]Select the values for sl and *m**for* (p=0:sl:1)
–Set$L_{p}=0$–*for* (k=1:m)–$\left[L_{p},{\left\{x_{k}^{i},w_{p,k}^{i}\right\}}_{i=1}^{N_{s}}\right]:=\mathrm{LL}\left[L_{p},{\left\{x_{k-N:k}^{i},w_{p,k-1}^{i}\right\}}_{i=1}^{N_{s}},y_{k}\right]$–end for–Initialization:*if*p=0–$L_{max}=L_{p}$and$\hat{p}=p$–Update:*else*–*If*$L_{p}>L_{max}$-$L_{max}=L_{p}$and$\hat{p}=p$end for

**Algorithm 4.** Online identification of latency probability.
[$L_{max},\hat{p}]:=\mathrm{ONLINE}[{\left\{x_{k-N:k}^{i},w_{k-1}^{i}\right\}}_{i=1}^{N_{s}},y_{1:k}$]


Select the value for sl

*for* (p=0:sl:1)
–
Set $L_{p}=0$
–
*for* (t=1:k)
–
$\left[L_{p},{\left\{x_{t}^{i},w_{p,t}^{i}\right\}}_{i=1}^{N_{s}}\right]:=\mathrm{LL}\left[L_{p},{\left\{x_{t-N:t}^{i},w_{p,t-1}^{i}\right\}}_{i=1}^{N_{s}},y_{t}\right]$

–
*end for*
–
Initialization: *if* p=0
–
$L_{max}=L_{p}$ and $\hat{p}=p$

–
Update: *else*
–
*If* $L_{p}>L_{max}$
-
$L_{max}=L_{p}$ and $\hat{p}=p$




*end for*



### 4.3. Computational Complexity

Attributing to the use of numerical search method to maximize the likelihood, the estimation of latency parameter is a computationally involved process. However, once the latency parameter is estimated, the computational cost of the proposed PF algorithm is not substantially higher than that of the standard PF. In the offline mode of identification, the proposed particle filter will run 1/sl times slower than the standard PF, where sl (0<sl<1) is the step length used for the search algorithm. That is, if the standard PF is $O(N_{s}n_{x}^{2})$ [42], then proposed PF in offline mode is $O(N_{s}n_{x}^{2}/sl)$. Similarly, for online identification, the modified particle filter will be $O(N_{s}n_{x}^{2}k)$, where *k* is the time step.

## 5. Simulation Results

To demonstrate the superiority of the proposed PF over the standard PF and the state-of-the-art algorithms for randomly delayed measurements, three different types of filtering problems are simulated. Although any general proposal density [39] can be chosen for the implementation of the proposed PF, in our simulations, the transitional prior density $P(x_{k}|x_{k-1})$ is used as the proposal density for its simplicity. The latency probability of delayed measurements is first identified by maximizing (36) over p∈[0,1] with the help of the proposed PF algorithm. Subsequently, the estimated probability *p* is used to implement the proposed PF for the given problems. To ensure a fair comparison of the proposed PF with the other state-of-the-art algorithms, two sets of simulation are carried out for each problem based on the measurement model used in the chosen filtering algorithm. In the first set of simulations, the proposed PF with estimated latency probability is compared against the standard PF and the cubature Kalman filter for the randomly delayed measurements (CKF-RD) [27] by using the randomly delayed measurements with packet drops. The proposed PF with other than the true value of *N* is also implemented to investigate the impact of selecting a wrong value for the maximum number of delay steps. The performances of all filters are compared in terms of the root mean square error (RMSE). Note that the proposed PF with the wrong value of *N* and the CKF-RD are implemented with the true value of the latency probability. In the second set of simulations, the proposed PF with the estimated latency probability is compared against the PF for multi-step randomly delay measurements (PF-MD) [29] by using the set of randomly delayed measurements with *no* packet drops. To demonstrate the importance of the latency estimation, PF-MD is implemented with both the true latency probability and the incorrect latency probability. Further, recognizing the practical limitation on the buffer-length and to avoid the large convergence error as discussed in Section 3.3, the true value of the maximum step delay is taken as N=2. However, the simulation work can be carried out with the larger value of *N* as shown for Problem 1. For brevity, the case with the larger *N* for other problems is not included in the paper.

MATLAB 2017a software is used to carry out the simulation work. No in-built subroutine for the PF is used and all the figures presented in this work are generated by running the standard instructions in MATLAB following the Algorithms 1–4. Both the truth and measurements are generated prior to filter implementation. The filtering algorithm uses the software generated measurement values for the PF to implement.

### 5.1. Problem 1

We consider a time-varying growth model that is widely used in literature because of its non-stationary property for validation of performances by nonlinear filters [9,23,43,44]. Nonlinear dynamics of the system are as follows:

(37) $$\begin{array}{cc} x_{k}=0.5x_{k-1}+ & 25\frac{x_{k-1}}{1+x_{k-1}^{2}}+8\cos\left(1.2k\right)+q_{k-1},\\ & z_{k}=x_{k}^{2}/20+v_{k}, \end{array}$$

where $q_{k-1}$ and $v_{k}$ are independent zero mean Gaussian processes with $E[q_{k}^{2}]=10$ and $E[v_{k}^{2}]=1$, respectively. The initial state $x_{0}∼N\left(0,1\right)$, and the received measurements, $y_{1:k}$ are generated by using (37) and (3) with N=2. The distribution of the initial estimated state $P\left({\hat{x}}_{0}\right)∼N\left(0,1\right)$ and the number of particles used for the simulation of this problem is, $N_{s}=1000$.

**Figure 2:**
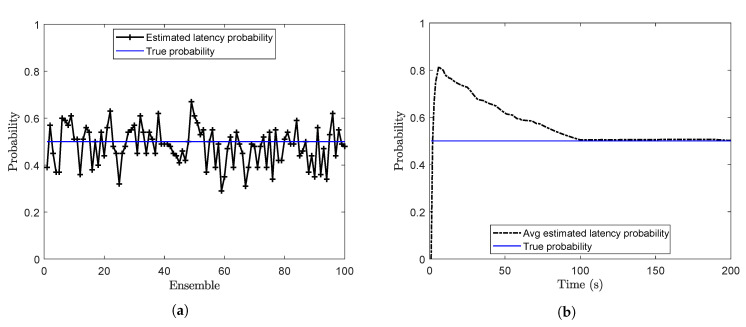
Estimation of the latency probability (**a**) Offline estimation (**b**) Online estimation (problem 1).

Offline estimation of the latency probability is carried out by using Algorithm 3 with sl=0.01 and m=500. The latency probability (*p*) at the end of each ensemble is calculated and plotted in Figure 2a. For this case, when the true value of *p* is 0.5, the mean of the estimated latency probability over 100 ensembles is calculated as 0.481. Online estimates of the latency probability are shown in Figure 2b. Here, the estimated probability at each time step is the running average of the estimated probabilities.

**Figure 3:**
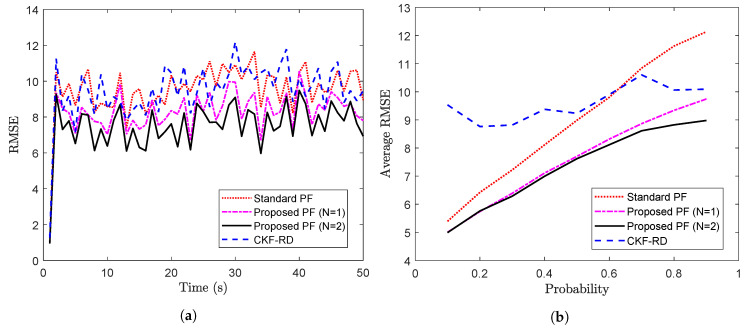
(**a**) RMSE vs. time steps considering p=0.5 (**b**) Avg RMSE vs. probability (Problem 1).

The proposed PF is implemented with N=1 and N=2 on the measurements as described above, and the results are compared against that of the CKF-RD and the standard PF. To compare the results, the RMSE calculated over 100 Monte Carlo (MC) runs are plotted over 50 time steps in Figure 3a. It can been seen that the proposed PF with N=2 outperforms the other three filters. Moreover, it is interesting to observe that the performance of the proposed PF with the wrong value of the maximum number of step delay (N=1) is closer to that of the proposed PF with N=2.

Further, the average RMSE calculated over 50 time steps for different values of true latency probability is shown in Figure 3b. As expected, for a higher probability value where packet drop is more likely, filter designed for N=2 performs better than the other filters.

**Figure 4:**
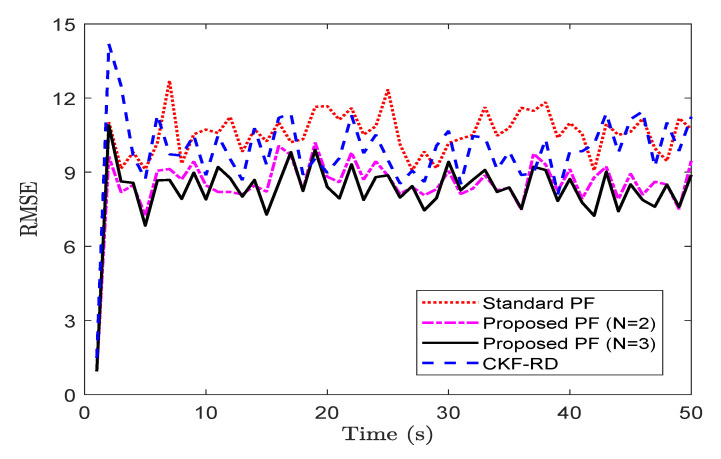
RMSE of different filters for N=3 and p=0.50 (problem 1).

To observe the impact on the performance of the proposed filter when the maximum number of step delay is increased, we take a case of N=3. Figure 4 shows the RMSE for the different filter while considering the true latency, p=0.50. The average RMSE of the proposed filter in Figure 3a with N=3 is 8.79 whereas that in Figure 4 with N=2 is 7.48. The increase in the length of memory for the proposed filter explains the increase in the RMSE.

**Figure 5:**
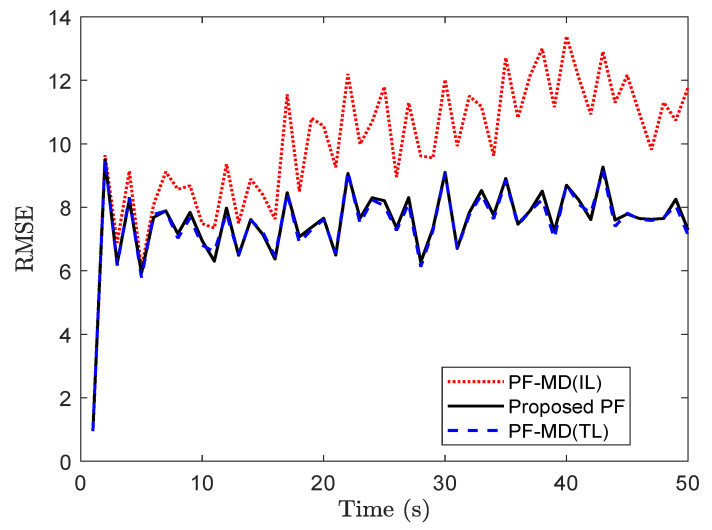
RMSE of different filters for measurements with no packet drops considering p=0.50 (problem 1).

For simulation with the set of randomly delayed measurements without any packet drops, the RMSE value for PF-MD with the true latency (PF-MD(TL)), the proposed PF and PF-MD with the incorrect latency (PF-MD(IL)) are plotted in Figure 5. It can be observed that the RMSE value of the proposed PF and PF-MD(TL) remain almost the same whereas the filtering performance of PF-MD degrades when the latency probability is unknown and considered an incorrect value.

Table 2 shows the comparison of the computational burden for the different filters. To make the computational comparison independent of the software and clock speed used for simulation, we have calculated the relative computational time for each filter. All computational time is calculated with respect to the computational time of the standard PF algorithm. Note that the given computational time for the proposed PF is after the estimation of the latency parameter. However, the estimation of the latency parameter requires 1/sl times running of the proposed PF algorithm.

### 5.2. Problem 2

Consider a ground surveillance problem where a moving target is tracked from a noisy sensor mounted on a moving platform at an approximately known altitude by using only bearing angle observations. This bearing-only tracking (BOT) problem has mainly two components, namely, the target kinematics and the tracking platform kinematics. A representative scenario is given in References [3,45]. The tracking platform motion may be represented by the following equations:

$$\begin{array}{c} \begin{array}{cc} x_{tp,k} & ={\bar{x}}_{tp,k}+\Delta x_{tp,k},\\ y_{tp,k} & ={\bar{y}}_{tp,k}+\Delta y_{tp,k},\;k=0,1,\cdots ,n_{step}, \end{array} \end{array}$$

where $x_{tp,k}$ and $y_{tp,k}$ represent the X and Y coordinates of the tracking platform at *k*th time-step, respectively. ${\bar{x}}_{tp,k}$ and ${\bar{y}}_{tp,k}$ are the known mean coordinates of the platform position and $\Delta x_{tp,k}$ and $\Delta y_{tp,k}$ are independent zero-mean Gaussian white noises with variances, $r_{x}=1\;m^{2}$ and $r_{y}=1\;m^{2}$, respectively. The mean values for position coordinates (in meters) are ${\bar{x}}_{tp,k}=4kT$ and ${\bar{y}}_{tp,k}=20$, where *T* is sampling time for the discretization expressed in seconds (s).

The target moves in X direction according to following discrete state space relations.

(38) $$x_{k}=Fx_{k-1}+Gq_{k-1},$$

where

$$x_{k}=\left[\begin{array}{c} x_{1,k}\\ x_{2,k} \end{array}\right],\;F=\left[\begin{array}{cc} 1 & T\\ 0 & 1 \end{array}\right],\;G=\left[\begin{array}{c} T^{2}/2\\ T \end{array}\right]$$

with $x_{1,k}$ and $x_{2,k}$ denoting the position (in m) and velocity (in m/s), respectively, of the target. $q_{k-1}$ is an independent zero-mean white Gaussian noise with variance $r_{q}=0.01\;m^{2}s^{4}$. The initial true states are assumed to be $x_{0}={\left[80\;1\right]}^{T}$.

The sensor measurement is given by

(39) $$z_{k}=z_{m,k}+v_{k},$$

where

$$z_{m,k}=h\left(x_{tp,k},y_{tp,k},x_{1,k}\right)=\arctan\frac{y_{tp,k}}{x_{1,k}-x_{tp,k}}$$

is the angle between the X axis and the line of sight from the sensor to target and $v_{k}$ is independent Gaussian white noise with zero mean and variance $r_{v}={\left(3^{\circ }\right)}^{2}$. For the implementation of CKF-RD, the measurement noise statistics, on account of the uncertainties in the position of the observer platform, need to be modified as given in Reference [3].

**Figure 6:**
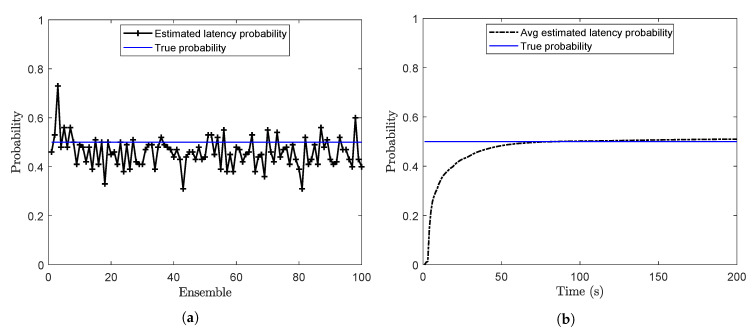
Estimated latency probability (**a**) Offline estimation (**b**) Online estimation (Problem 2).

The randomly delayed measurements with packet drops, $y_{1:k}$, are generated for 21s by using (3) and (39) with N=2. The number of particles used for approximating the pdf is $N_{s}=3000$. At the beginning of simulation, the latency probability of received measurements is identified offline with T=0.05 s, m=400, and sl=0.01. Latency probability at the end of each ensemble is calculated. The mean value of estimated *p* over 100 ensembles is calculated as 0.460, whereas its true value is 0.5. For online identification, *T* is taken as 0.1 s and the running average of latency probability is calculated at each time step. The plots for the offline and online estimations are shown in the Figure 6a,b, respectively.

**Figure 7:**
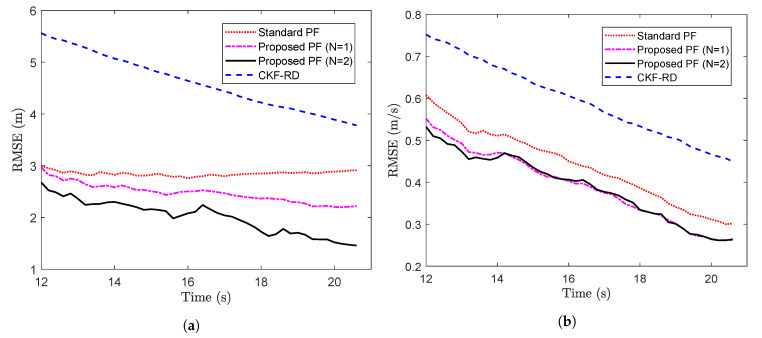
RMSE vs. time steps with p=0.5. (**a**) State $x_{1,k}$ (**b**) State $x_{2,k}$ (Problem 2).

Now, the proposed PF with N=1 and N=2 are implemented with the true and estimated latency probabilities, respectively. The results of CKF-RD and the standard PF for same set of delayed measurements are compared against that of proposed PF. The RMSE of four filters with the true latency probability p=0.5 and sampling time T=0.2 s, which have been calculated over 100 MC runs, are plotted in Figure 7a,b. From the plots, it can be seen that using a filter designed for delayed measurements is a better choice than a standard PF where the delays are not accounted.

**Figure 8:**
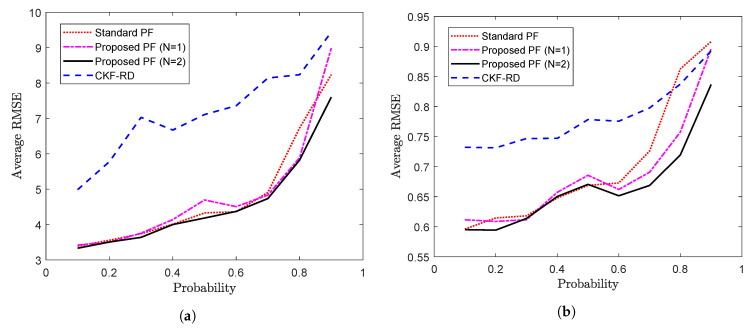
Average RMSE vs. probability (**a**) State $x_{1,k}$ (**b**) State $x_{2,k}$ (Problem 2).

Further, the average RMSE is calculated over 105 time steps for the different values of *p*. The average RMSE for two states, $x_{1,k}$ and $x_{2,k}$ are plotted in Figure 8a,b respectively. It can be seen that the difference in performance of the proposed PF and the standard PF becomes pronounced as the value of probability increases. However, at higher probabilities, the performance of the proposed filter also gets deteriorated on account of the higher rate of the packet loss.

**Figure 9:**
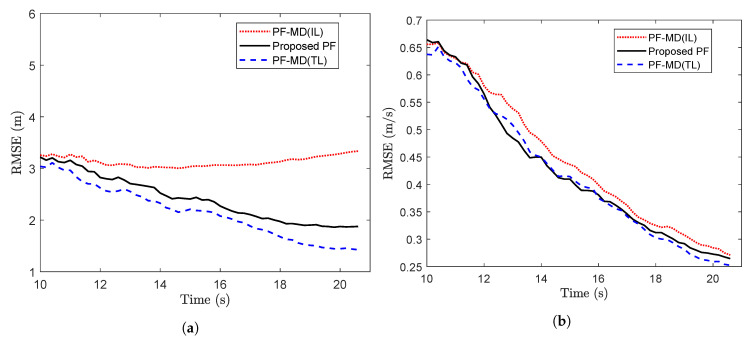
RMSE of different filters for measurements with no packet drops considering p=0.5. (**a**) State $x_{1,k}$ (**b**) State $x_{2,k}$ (Problem 2).

To compare the performance of the proposed PF with the estimated latency, and PF-MD with the unknown latency, randomly delayed measurements with no packet drops are generated using the measurement model of Reference [29]. The RMSE for three filters are plotted in Figure 9. It can be observed that estimation of the latency affects the filtering process and the use of its incorrect value degrades the performance of a filter. The relative computational burden is given in the Table 3.

### 5.3. Problem 3

Consider a coordinated turn model for an aerospace target tracking problem for an aircraft that executes a maneuvering turn in a two-dimensional plane with a fixed, but unknown turn rate Ω. This uses the bearing and the range measurement observed from a radar to estimate the kinematics of the target. The five-dimensional state vector for the kinematics of aircraft is considered as $x_{k}={\left[\zeta \;\dot{\zeta }\;\eta \;\dot{\eta }\;\Omega \right]}^{⊤}$, where ζ and η represent positions, and $\dot{\zeta }$ and $\dot{\eta }$ represent velocities along the *X* and *Y* co-ordinates, respectively. The discrete-time state dynamics of the aircraft is given as [46]:

(40) $$\begin{array}{c} x_{k}=\left[\begin{array}{ccccc} 1 & \frac{\sin\Omega T}{\Omega } & 0 & -\left(\frac{1-\cos\Omega T}{\Omega }\right) & 0\\ 0 & \cos\Omega T & 0 & -\sin\Omega T & 0\\ 0 & \frac{1-\cos\Omega T}{\Omega } & 1 & \frac{\sin\Omega T}{\Omega } & 0\\ 0 & \sin\Omega T & 0 & \cos\Omega T & 0\\ 0 & 0 & 0 & 0 & 1 \end{array}\right]x_{k-1}+q_{k-1}, \end{array}$$

where *T* is the time between two successive measurements. $q_{k-1}$ is a zero mean Gaussian noise with covariance $Q=\mathrm{diag}[q_{1}M\;q_{1}M\;q_{2}T]$, where $q_{1}$ and $q_{2}$ are the noise intensity parameters, and $M=\left[\begin{array}{cc} \frac{T^{3}}{3} & \frac{T^{2}}{2}\\ \frac{T^{3}}{2} & T \end{array}\right]$. The range, *r*, and bearing, θ are the measurement available for target tracking, which are being measured by a radar placed at the origin. The noise-corrupted measurements can be expressed as

(41) $$z_{k}={\left[r_{k}\;\theta_{k}\right]}^{⊤}={\left[\sqrt{\zeta_{k}^{2}+\eta_{k}^{2}}\;\tan^{-1}\left(\frac{\eta_{k}}{\zeta_{k}}\right)\right]}^{⊤}+v_{k},$$

where $v_{k}$ is an independent zero-mean Gaussian noise with covariance $R=\mathrm{diag}[\sigma_{r}^{2}\;\sigma_{\theta }^{2}]$. Data for the different parameters used in this simulation are given in Table 4. Initial values for the state and covariance are $x_{0}=[1000\;$ m $\;300\;ms^{-1}\;1000\;$ m $\;0\;{\mathrm{ms}}^{-1}$$-3^{\circ }s^{-1}{]}^{T}$ and $P_{0|0}=\mathrm{diag}\left[100\;m^{2}\;10\;m^{2}s^{-2}\;100\;m^{2}\;10\;m^{2}s^{-2}\;100\;{\mathrm{mrad}}^{2}s^{-2}\right]$, respectively.

**Figure 10:**
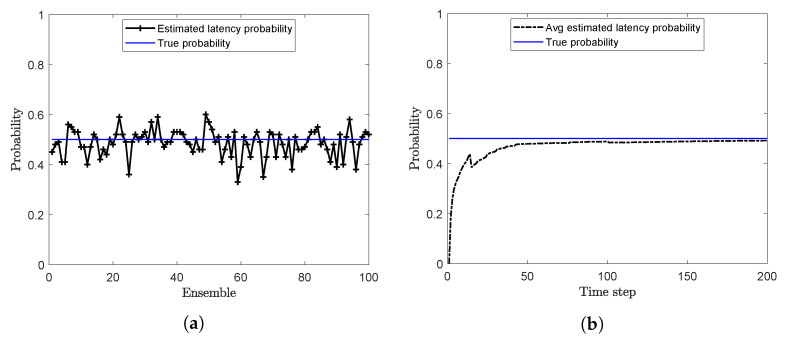
Estimated latency probability (**a**) Offline estimation (**b**) Online estimation (Problem 3).

The randomly delayed measurements with packet drops, $y_{1:k}$, are generated by using (41) and (3) with N=2 and a probability, p=0.5. The number of particles used for approximating the pdf is $N_{s}=8000$. In the beginning of simulation, the latency probability of received measurements is identified offline with m=200 and sl=0.01. The latency probability at the end of each ensemble is calculated and plotted in Figure 10a. The mean value of the estimated probabilities $\hat{p}$ over 100 ensembles is calculated as 0.4864 against its true value 0.5. Figure 10b shows the online estimation of latency probability.

**Figure 11:**
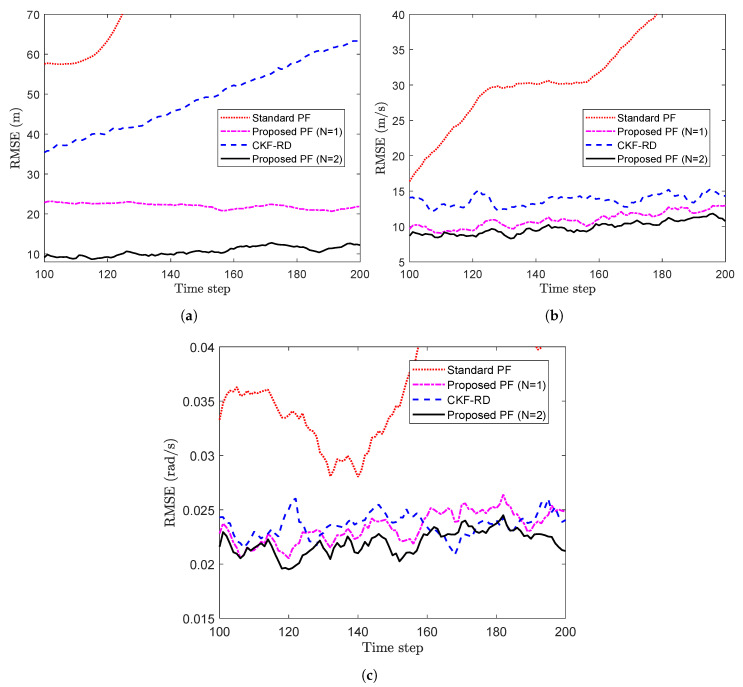
RMSE vs. time steps considering p=0.5. (**a**) RMSE of position (**b**) RMSE of velocity (**c**) RMSE of turn rate (Problem 3).

To track the maneuvering target, the proposed PF with N=2 and N=1 (a wrong value of the maximum number of delay steps) are implemented. To demonstrate the superiority of the proposed PF, simulation results of the CKF-RD and the standard PF for the same set of measurements and with the true value of latency probability, are compared against that of the proposed PF. The RMSE of the position, velocity and turn rate are the performance metrics as defined in Reference [46]. The RMSE of position, velocity and turn rate for the four filters with p=0.5, are plotted in Figure 11a–c, respectively. Since the standard PF does not consider the correlation of current measurement with previously observed states due to random delays, it diverges, particularly in case of the RMSE in position. It can also be observed in the RMSE plots that the proposed PF with N=1, which has accounted at least partially for the presence of random delays, performs better than the standard PF. This is significant since the maximum number of delay steps will have to be decided by the user and setting N=1 might still yield some benefit over assuming no delay. Note that the difference in RMSE of position between the proposed PF and the standard PF or, the CKF-RD is considerable and is worth the relatively higher computational cost paid to obtain it.

**Figure 12:**
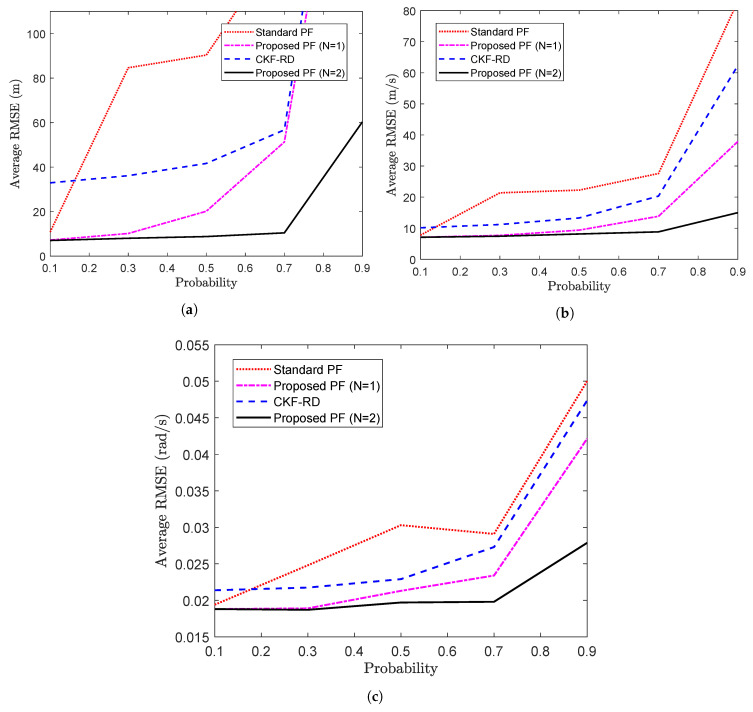
Average RMSE vs. probability (**a**) Average RMSE of position (**b**) Average RMSE of velocity (**c**) Average RMSE of turn rate (Problem 3).

Further, to observe the impact of latency probability on the performances of the different filters, the time-averaged value of the RMSEs of the position, velocity and turn rate are plotted against different values of the *p* in Figure 12a–c, respectively. It can be seen that with high value of probability, that is, when the random delays are more likely, the standard PF and proposed PF with N=1 become significantly less applicable than the proposed PF with N=2. It can also be observed that when the probability is high with insufficiently higher value of *N*, the rate of packet drops increases and even the performance of the proposed PF (N=2) gets deteriorated.

**Figure 13:**
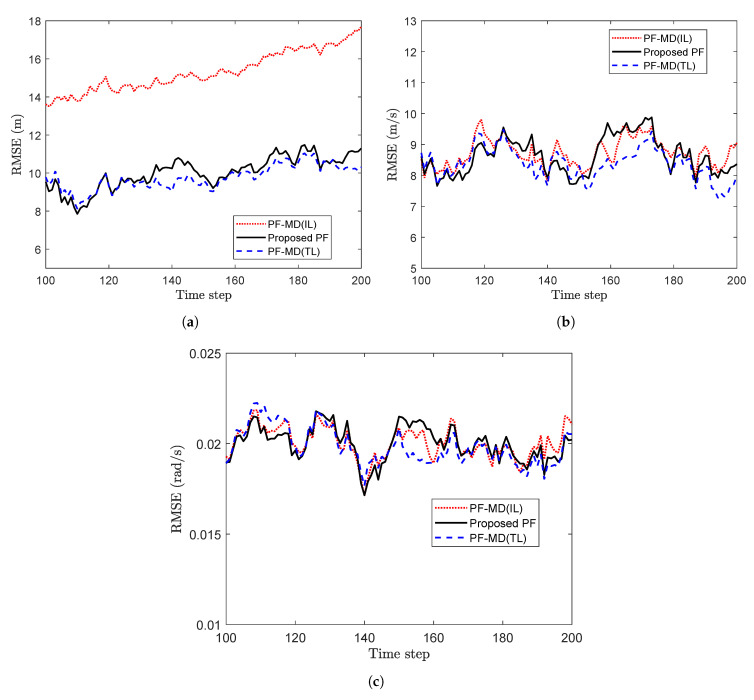
RMSE of different filters for measurements with no packet drops considering p=0.5. (**a**) RMSE of position (**b**) RMSE of velocity (**c**) RMSE of turn rate (Problem 3).

The performance of the proposed PF and PF-MD is compared in terms of the RMSE in Figure 13. To be in synchronization with the model of Reference [29], the measurements used for the estimation are without any packet drops. It can be observed from the plot that without the knowledge of latency, the performance of PF-MD degrades considerably, which suggests estimation of the latency is needed as it is presented in the proposed PF. Table 5 shows the relative computational burden for different filters.

## 6. Conclusions and Discussion

The standard PF loses its applicability if measurements are randomly delayed at the receiver. The random delays are possible in a system where the sensor and the receiver are connected through a network with bandwidth limitation.

In this paper, a recursion equation of the importance weight was developed to handle delays in measurements. A practical measurement model based on i.i.d. Bernoulli random variables, which includes the possibility of random delays in receiving the observations along with the packet drop situation if any measurement suffers a delay more than the data buffer length, was adopted. Moreover, the latency probability of the received measurements is usually unknown in practical cases. Hence, this paper presented a method to identify the latency probability in the randomly delayed measurement environment. Further, this paper explored the conditions to ensure the convergence of the proposed PF and the trade-off in selecting the maximum delay.

To validate the performance of the proposed filter and demonstrate its superiority, three numerical examples are simulated using the standard PF, the CKF-RD, the proposed PF with wrong selection of maximum delay and the proposed PF with the correct value of maximum delay. Simulation results show that a filter designed for the delayed measurements, even considering less delay than the actual, performs better than the standard PF. If the random delay is more likely in a system, number of the maximum possible delay should be chosen such that it strikes a balance between avoiding the information loss and minimizing the convergence error. Further, the proposed PF is compared with PF-MD, which is designed for the multi-step randomly delayed measurements without any packet drops and assumes that the latency probability is known. Simulation results showed that the performance of a filter depends on the correct value of latency and its estimation is necessary for the better accuracy of the filtering process.

## Figures and Tables

**Table 1:** Features comparison for proposed work and previous works.

Work	Random Delays	Packet Drops	Latency Estimation	Filtering
[28]	Single step	×	✓	SMC
[29]	Multi-step	×	×	SMC
[30]	Single-step	×	✓	Gaussian
[31]	Single-step	×	✓	Gaussian
Proposed work	Multi-step	✓	✓	SMC

**Table 2:** Relative computational time for different algorithms.

Algorithms	Relative Computational Time
CKF-RD	0.42
Standard PF	1
PF-MD	1.59
Proposed PF	1.65

**Table 3:** Relative computational time for different algorithms.

Algorithms	Relative Computational Time
CKF-RD	0.053
Standard PF	1
PF-MD	1.285
Proposed PF	1.330

**Table 4:** Parameters used in tracking

Sampling Time (*T*)	0.125 s
Turn Rate (Ω)	$-3^{\circ }s^{-1}$
$q_{1}$	$0.1\;m^{2}s^{-3}$
$q_{2}$	$1.75\times 10^{-4}\;s^{-3}$
$\sigma_{r}$	10 m
$\sigma_{\theta }$	$\sqrt{10}$ mrad

**Table 5:** Relative computational time for different algorithms.

Algorithms	Relative Computational Time
CKF-RD	0.025
Standard PF	1
PF-MD	1.512
Proposed PF	1.610

## Abbreviations

The following abbreviations are used in this manuscript:

pdf	Probability density function
PF	Particle filter
EKF	Extended Kalman filter
UKF	Unscented Kalman filter
EM	Expectation-Maximization
SMC	Sequential Monte Carlo
SIR	Sequential importance resampling
LL	Log-Likelihood
CKF-RD	Cubature Kalman filter for randomly delayed measurements
PF-MD(IL)	Particle filter for multiple delayed measurements with incorrect latency
PF-MD(TL)	Particle filter for multiple delayed measurements with true latency
RMSE	Root mean square error
BOT	Bearing-only tracking
i.i.d.	Independently and identically distributed
